# Biomaterials and Extracellular Vesicle Delivery: Current Status, Applications and Challenges

**DOI:** 10.3390/cells11182851

**Published:** 2022-09-13

**Authors:** Kasey S. Leung, Sajjad Shirazi, Lyndon F. Cooper, Sriram Ravindran

**Affiliations:** 1 Department of Oral Biology, College of Dentistry, University of Illinois Chicago, Chicago, IL 60612, USA; 2 School of Dentistry, Virginia Commonwealth University, Richmond, VA 23298, USA

**Keywords:** mesenchymal stem cells, extracellular vesicles, biomaterials, tissue repair, scaffolds, biopolymers

## Abstract

In this review, we will discuss the current status of extracellular vesicle (EV) delivery via biopolymeric scaffolds for therapeutic applications and the challenges associated with the development of these functionalized scaffolds. EVs are cell-derived membranous structures and are involved in many physiological processes. Naïve and engineered EVs have much therapeutic potential, but proper delivery systems are required to prevent non-specific and off-target effects. Targeted and site-specific delivery using polymeric scaffolds can address these limitations. EV delivery with scaffolds has shown improvements in tissue remodeling, wound healing, bone healing, immunomodulation, and vascular performance. Thus, EV delivery via biopolymeric scaffolds is becoming an increasingly popular approach to tissue engineering. Although there are many types of natural and synthetic biopolymers, the overarching goal for many tissue engineers is to utilize biopolymers to restore defects and function as well as support host regeneration. Functionalizing biopolymers by incorporating EVs works toward this goal. Throughout this review, we will characterize extracellular vesicles, examine various biopolymers as a vehicle for EV delivery for therapeutic purposes, potential mechanisms by which EVs exert their effects, EV delivery for tissue repair and immunomodulation, and the challenges associated with the use of EVs in scaffolds.

## 1. Introduction

The use of extracellular vesicles (EVs) in lieu of stems cells in scaffolds has become increasingly popular in recent years. Stem cells such as mesenchymal stem cells (MSCs) have immunomodulatory and differentiation effects, however, they have been found to cause abnormal differentiation and tumor formation [1]. MSCs exert their therapeutic functions via their secretome, including EVs [2]. For tissue engineering, EVs are an attractive alternative to stem cell transplantation as they have emerged as important mediators of cellular communication and can directly affect a number of biological processes in target cells [3]. Thus, regenerative research is shifting from the use of stem cells to the use of EVs.

Scaffolds serve as an approach to restore form and function to diseased, damaged, and lost tissue by acting as the ECM that supports the cells and their fate and function [4]. Various natural and synthetic biopolymers can be used to create such scaffolds. Incorporating EVs into the synthesis of scaffolds provides a system that supports host regeneration through structural and physiological means. In this review, we discuss EV integration into different types of biopolymers for a variety of therapeutic applications. Additionally, we briefly highlight the potential of using EV-functionalized scaffolds for tissue repair in different organs systems as well as the possible roles EVs play in immunomodulation. Finally, we examine the challenges in EV loading, integrity, delivery, and scaling up.

## 2. Extracellular Vesicles (EVs)

**Figure 1:**
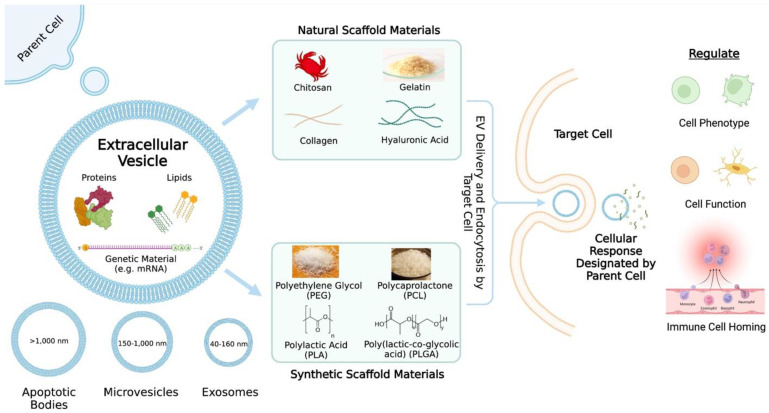
An overview of extracellular vesicle (EV) delivery via scaffolds. The contents and categories of the extracellular vesicles as well as what the extracellular vesicles may regulate are described. Common natural and synthetic biomaterials for scaffold fabrication are highlighted.

Extracellular vesicles are nanosized cell-derived membranous structures and can be categorized into several subclasses including exosomes (40–160 nm), microvesicles (150–1000 nm), and apoptotic bodies (>1000 nm) [5] (Figure 1). Exosomes are formed by the inward budding of endosomes and results in the generation of intraluminal vesicles within multivesicular bodies (MVBs) [6]. When MVBs fuse with the plasma membrane, the intraluminal vesicles are released into the extracellular space and are then referred to as exosomes [6]. It is unlikely that researchers will be able to capture live images of EV release in order to assign EVs to subclasses [7]. Thus, the authors often refer to EV subclasses based on the physical characteristics (e.g., small EVs (sEVs)), biochemical composition (e.g., Annexin A5-stained EVs), conditions (e.g., hypoxic EVs), or cell of origin (e.g., hMSC EVs) [7]. Ultimately, it is important to continue studying specific and reliable markers of EV subtypes, so that a consensus regarding nomenclature can be established.

EV isolation poses several challenges. For instance, the complete isolation of EVs from other entities, such as proteins and RNA granules, is unlikely [7,8]. Furthermore, there is no consensus on the best method for EV isolation as well as to how pure an EV preparation should be, as this depends on the experimental question and the purpose of the EVs [7,9]. For example, therapeutic applications in which function is most important may not require highly purified EVs, whereas attributing a biomarker to vesicles will likely require highly purified EVs [7]. Nevertheless, researchers are able to isolate EVs from other non-EV components as well as different types of EVs from each other to various degrees using different available techniques such as ultracentrifugation and size-exclusion chromatography [7,10,11,12,13].

A major issue regarding EV characterization is that a relatively high number (17%) of published EV-related articles do not provide EV characterization, and about half of the EV-related articles (55%) only used an antibody-based assay to detect EV proteins [14]. EV characterization is necessary to assess different isolation methods and establish that biomarkers or functions are associated with EVs rather than other co-isolated substances [7]. Examples of EV characterization and quality-control methods include particle tracking, Western blotting, electron microscopy, RNA profiling, and advanced cytometry [15,16]. Thus, it is crucial that the authors include EV characterization in their studies, whether that be by providing EV quantification, protein composition, single vesicle analysis, topology of EV-associated components, or other means [7].

EVs play roles in many physiological and pathological processes [5,17] including suppressing inflammatory responses [18,19], modulating cellular function and regenerating tissue injuries [20,21], and modulating the immune system [22]. Once endocytosed by target cells, EVs trigger a cellular response designated by the parental cell to the target cell [20]. In other words, the roles that EVs fulfill depend on their origin [23], and when taken up by target cells, EVs release their contents to enact changes in gene expression (Figure 1) [24]. The cargo EVs carry include proteins, lipids, and genetic material (e.g., miRNA) [23]. Studies have investigated specific EV cargoes that are related to positive therapeutic outcomes. These studies will be discussed further in the following sections. Ultimately, EV contents allow for the regulation of phenotype, function, and immune cell homing, highlighting the potential benefits of using EVs in bioengineering [25].

## 3. Scaffolds as a Solution for EV Delivery

Despite the therapeutic potential of EVs, they have short-lived effects when administered alone. It has been shown that systemically administered EVs have a short half-life and are quickly cleared from the body, making it difficult to realize the long-term effects of EV treatment [26,27]. As a result, the therapeutic effect of EVs is minimal. One solution to this issue is the utilization of scaffolds for the localized delivery and controlled release of EVs. Scaffolds can contain EVs and deliver them to sites of interest over time, thus optimizing the EV therapeutic potential. Furthermore, scaffolds provide a proper environment for tissue regeneration by providing space for host cell migration, mechanical support and integrity, and even cell signaling via bioactive molecules (e.g., EVs). Physiologically active EVs interact with surrounding ECM molecules [28]. Thus, it is important that scaffolds mimic the ECM complex to support active-EV delivery. By applying EV-laden scaffolds to injury sites, the EVs in the scaffold are protected and released from the scaffold in a sustained manner [29]. Released EVs can communicate with endogenous cells and extracellular components, which allows them to play a role in therapeutic effects such as the remodeling process [29]. Overall, scaffolds bridge the therapeutic benefits of extracellular vesicles and clinical application by creating an appropriate environment for EV delivery.

## 4. Biopolymers as Delivery Systems

There are two main types of biopolymers used in bioengineering: natural and synthetic biopolymers (Figure 1). Natural biomacromolecules that have been studied include silk fibroin, collagen, gelatin, chitosan, and hyaluronic acid. Widely studied synthetic biopolymers for scaffolds include polyethylene glycol (PEG), polycaprolactone (PCL), poly(lactic-co-glycolic acid) (PLGA), and poly(L-lactide) (PLLA). Each type of biomaterial comes with benefits and challenges. For example, natural biomaterials are inherently variable depending on the biological source, may have unwanted biological residues, and there may be issues concerning mechanical stability [30]. Meanwhile, synthetic biomaterials lack native tissue topography and structure that is more typical of natural biomaterials and may contribute to a toxic environment due to chemical crosslinking [31]. In general, immunogenicity, limited mechanical properties, and the lack of consistency are always areas of concern when it comes to engineering scaffolds [32]. Additionally, issues that may arise with the use of cell-based technology, such as EVs, include regulatory hurdles in terms of safety and clinical translation (e.g., mutated cell DNA and off-the-shelf-storage, respectively) [33]. Nevertheless, biopolymer-based scaffolds remain one of the most widely used methods for the delivery of biological signals, especially in the field of tissue engineering. In the following sections, we discuss the natural and synthetic biopolymers frequently used for scaffold fabrication and the therapeutic effects of incorporating EVs into these scaffolds.

## 5. Natural Biopolymer Scaffolds for Therapeutic EV Delivery

Natural biopolymers such as alginate, silk fibroin, collagen, gelatin, and chitosan have numerous advantages as scaffold materials. They all mimic the natural ECM and are also biocompatible, biodegradable, and cause fewer immunogenic reactions [34,35,36]. Furthermore, these natural biopolymers can promote cell adhesion, migration, and differentiation function, as well as produce non-cytotoxic degradation products that can be removed via metabolic pathways in vivo [34]. Thus, natural biopolymers offer select advantages for engineering scaffolds for therapeutic applications. Enhancing the natural biopolymer scaffolds with EVs has shown positive therapeutic effects regarding a myriad of tissues and diseases (Table 1). Below, we highlight examples of factors that have been delivered with commonly studied natural biopolymers as well as several studies that utilize EV-laden natural biopolymer scaffolds for various therapies.

### 5.1. Sodium Alginate

Sodium alginate (SA) is a linear polysaccharide derived from brown seaweed [55]. More specifically, SA is a derivative of alginic acid and is composed of α-1-guluornic (G) and 1,4-linked-β-D-mannuronic (M) monomers [55]. Alginate has been used to deliver various classes of drugs including NSAIDs [56], chemotherapeutics [57,58], and anesthetics [59]. Hormones such as insulin [60] and salmon calcitonin [61] have also been delivered via alginate. Furthermore, neuropeptides [62], genetic material [63], and probiotics [64] have been delivered using alginate.

EV-laden alginate-based hydrogels have been studied for a wide range of therapeutic applications including diabetic wound healing [37], peripheral nerve regeneration [38], and myocardial infarction (MI) [26]. Lv et al. delivered bone marrow mesenchymal stem cell (MSC)-derived small EVs (sEVs) to the heart using a natural sodium alginate hydrogel as a therapy for myocardial infarction. The hydrogels were embedded with sEVs by simply mixing the sEV solution with the sodium alginate solution prior to hydrogel formation with calcium chloride solution [26]. Additionally, the authors found that hydrogels formed with 0.5% or 1% calcium chloride solutions resulted in nearly all of the sEVs being released by day 10 with a quick burst of sEV release during the first couple of days compared to a 2% calcium chloride solution [26].

Lv et al. reasoned that hydrogels with a quicker sEV release profile would better suit a myocardial infarction model [26]. The authors labeled sEVs and found through ex vivo imaging that sEVs embedded in the hydrogel (sEV-gel) were retained in the heart compared to freely injected sEVs [26]. Lv et al. examined the expression levels of miRNAs (miR) related to anti-apoptosis and pro-angiogenesis and performed TUNEL staining; they found that treatment using the sEV-gel showed decreased cardiac cell apoptosis [26]. Additionally, Lv et al. looked at the number of CD68+ macrophages as well as the ratio of CD206+ to CD68+ macrophages and found that the sEV-gel treatment promoted M1 to M2 macrophage polarization only a couple days after myocardial infarction [26]. Furthermore, Lv et al. used CD31+ staining, α-SMA staining, and Western blotting to show that sEV-gel treatment promoted angiogenesis [26]. Using echocardiography and histology, Lv et al. saw that the sEV-gel treatment resulted in improved cardiac function and enhanced scar thickness compared to only using sEVs [26].

Overall, the authors demonstrated that the sEV-gel treatment promoted angiogenesis, reduced cardiac apoptosis and fibrosis, and improved cardiac function after MI [26]. This study highlights the importance of using a scaffold to allow for an appropriate, efficient, and locally concentrated delivery of EVs, for without some type of retention system, administered EVs will be quickly cleared by the body and will have a minimal therapeutic effect.

### 5.2. Silk Fibroin

Silk fibroin (SF) is a hydrophobic protein from the *Bombyx mori* silkworm that self-assembles into strong and resilient materials [65,66]. Silk fibroin on its own is biocompatible, has controllable biodegradability, and has tunable mechanical properties [67]. It also causes minimal inflammation of host tissue, is low-cost, and easy to use [67]. Anti-proliferative [68], anti-inflammatory [69,70], anabolic [70], and anti-retroviral drugs [71] have been delivered using silk fibroin. Additional drugs include anti-inflammatory compounds such as curcumin [72,73] and chemotherapeutics [74,75]. Aside from drug delivery, SF has been utilized for the delivery of antibodies [76], proteins [76,77], hormones (e.g., insulin) [78], genetic material [79], and cells (e.g., mesenchymal stem cells) [80].

Cunnane et al. examined the effect of human adipose-derived mesenchymal stem cell EVs (hADMSC EVs) on vascular cells in vitro [33]. They found that the application of these EVs on smooth muscle cells and endothelial cells increased proliferation as well as migration in a dose-dependent manner [33]. Cunnane et al. then vacuum-seeded EVs into porous silk-based tubular scaffolds by turning mounted scaffolds within a vacuum chamber and infusing the scaffolds with an EV isolate. Using a micro bicinchoninic acid protein assay and fluorescent imaging, they found that this method of seeding retained a greater amount of protein and increased EV coverage, respectively, within the scaffold compared to the soak-loading method [33].

Cunnane et al. implanted the silk-based scaffolds into rat aortas to study the remodeling capacity of the EV-doped scaffolds. After 8 weeks, the explants were stained for cell, collagen, and elastin distribution [33]. Additionally, elastin and collagen content assays were performed to quantify protein deposition within each explant [33]. Their in vivo findings showed that the inclusion of EVs in the scaffold wall improved patency and matrix deposition, including more elastin and collagen production, which is crucial for neo-tissue formation [33]. This study demonstrates that EVs play an effective bioinstructive role when incorporated into and delivered by SF-based vascular grafts.

### 5.3. Chitosan

Chitosan is a cationic polysaccharide derived from chitin and is made up of diglucose amine and N-acetyl glucose amine groups [81,82,83]. Antibiotics [81,83], antivirals [84], and immunosuppressants [85] have been delivered using chitosan. Additionally, chitosan has been utilized to deliver insulin [82] and genetic material [86,87,88].

Chitosan-based scaffolds have been used to deliver EVs to improve bone defect repair [41], corneal diseases [42], skin wound healing [32], and articular cartilage injuries [43]. Wu et al. developed chitosan-based thermosensitive hydrogels laden with bone mesenchymal stem cell (BMSC)-derived sEVs to accelerate osteogenesis and angiogenesis. After isolating the sEVs, Wu et al. characterized the sEVs through analyzing the size distribution and morphology of the sEVs as well as through Western blotting to detect sEV-specific surface markers [41]. The authors also ensured that the sEVs could be internalized by BMSCs and HUVECs [41]. In vitro experimentation included examining the ALP activity of BMSCs when exposed to sEVs. The results indicated the early-stage osteogenic differentiation of BMSCs when exposed to sEVs, shown by elevated ALP activity in the sEV groups [41]. Furthermore, Wu et al. found through examining the mRNA (*OCN, OPN,* and *Runx2*) and protein (OCN, OPN, and RUNX2) levels that BMSC-sEVs can upregulate osteogenic gene expression [41]. The authors also studied the migratory capability of HUVECs exposed to sEVs and found that the proliferation of HUVECs exposed to sEVs increased compared to the control group [41]. Furthermore, mRNA and protein levels relevant to angiogenesis increased in cells exposed to sEVs [41]. Wu et al. added β-glycerphosphate to chitosan to formulate a thermosensitive injectable hydrogel, and they found that sEVs embedded in this hydrogel showed a good slow-release performance of 80% sEV release on day 8, with a slowed release rate thereafter [41].

The in vivo micro-CT results of a calvarial defect model showed that hydrogels embedded with sEVs resulted in a greater area of newly formed bone compared to other groups [41]. Histological staining showed that there was newly formed bone in the sEV-hydrogel and hydrogel-only groups compared to the control group, which resulted in a defect mainly filled with fibrotic connective tissue [41]. Immunohistochemical staining of bone defect sections indicated more CD31+ in the sEV-hydrogel group compared to the hydrogel-only group, which indicated new vessel formation within the bone defect [41]. Overall, the in vivo experimentation demonstrated that sEVs promote calvarial defect repair and enhanced osteogenesis and angiogenesis [41].

Wu et al. examined the potential cause of their observed results by studying the relationship between miR-21 and SPRY2. After performing the reporter assays and rescue experiments, they found that the angiogenic protein levels in cells transfected with miR-21 mimics were higher than the control cells [41]. These experiments indicated that exosomal miR-21 may promote HUVEC migration and angiogenesis by targeting SPRY2 [41].

Overall, Wu et al. successfully developed a thermosensitive injectable chitosan-based hydrogel laden with BMSC-derived sEVs. The hydrogel promoted bone healing and served as a scaffold for sEVs [41]. The sEV-loaded hydrogel promoted bone healing in vivo by enhancing angiogenesis, which may be mediated by miR-21 expression upregulation in sEVs and the regulation of SPRY2 by miR-21 [41]. This study highlights the potential of delivering EVs via scaffolds to promote bone regeneration and the importance of understanding the mechanism behind positive results to continue improving the therapeutic outcomes.

### 5.4. Collagen

Collagen is the most abundant protein in mammals [89] and is composed of three intertwined α-chains [90]. Some of the functions of collagen include cell adhesion and migration, tissue repair, and scaffolding [91]. Collagen has proven to be useful in medical applications as a delivery tool. Similar to the natural biopolymers discussed thus far, collagen has been utilized to deliver drugs [92,93], cells [94,95], and bioactive substances with antioxidant properties [96]. It is also possible to deliver growth factors using collagen [97,98].

Collagen-based scaffolds have been utilized for therapeutic purposes including bone regeneration [39] and endometrium regeneration [40]. Xin et al. designed a collagen scaffold containing umbilical cord-derived mesenchymal stem cell (UC-MSC)-derived exosomes for endometrial regeneration in a rat endometrium-damage model [40]. The authors confirmed successful exosome extraction through TEM, NTA, and Western blot [40]. Xin et al. added exosome suspension dropwise to the collagen scaffold, and they obtained a sustained release profile with a majority of exosomes being released within 14 days [40].

Xin et al. examined short-term and long-term outcomes from a rat endometrium-damage model involving exosome-collagen, collagen-only, exosome-only, and no treatment (control) groups [40]. Through H&E staining, Xin et al. found that transplantation of the exosome-collagen scaffold promoted endometrium regeneration and glandular reconstruction, which was related to rapid cell proliferation and re-epithelialization [40]. Additionally, Xin et al. performed immunostaining and Masson’s trichrome staining and found that transplantation of the exosome-collagen resulted in excellent neovascularization, reduced fibrosis formation, and promoted collagen remodeling [40]. Through immunohistochemical staining and subsequent software analysis, Xin et al. found that a high number of anti-estrogen receptor α (ERα) positive cells and anti-progesterone receptor (PR) positive cells were present 30 days after exosome-collagen treatment, which suggests the rapid functional recovery of the regenerated endometrium [40]. Furthermore, Xin et al. showed through Evans blue staining that the implantation of the exosome-collagen scaffold resulted in the structural and functional reconstruction of endometrium that could support implantation and the development of embryos in vivo [40].

Xin et al. investigated the potential mechanisms behind the promising outcomes observed with exosome-collagen treatment. The authors found that exosome-collagen treatment promoted the macrophage infiltration within 7 days, with high numbers of M1 macrophages in the exosome-collagen and collagen-only groups within 3 days, likely due to the immunogenicity of the implanted collagen scaffold [40]. Additionally, the exosome-collagen group showed the highest numbers of M2 macrophages within 7 days, indicating that exosome incorporation may have induced the polarization of macrophages to the M2 phenotype [40]. Furthermore, Xin et al. examined the M1 and M2 macrophage-related cytokines. When looking at the M1-related cytokines, they found that the inclusion of exosomes in the scaffold eased inflammation due to the foreign body reaction to collagen within 7 days [40]. Furthermore, there was an enhanced expression of M2-related cytokines with exosome-collagen transplantation. Altogether, transplantation of the exosome-collagen scaffold promoted M1 macrophage infiltration during the early stages of wound healing and induced macrophage transition from an M1 to a M2 phenotype during a later stage of healing [40].

Through analyzing their RNA-Seq data against a publicly-available database, Xin et al. identified a top candidate related to macrophage immunomodulation (miR-223-3p), which has been reported to promote macrophage polarization to an M2 phenotype, as the key cargo within exosomes [40]. Xin et al. believe that miR-223-3p may target *Stmn1* within macrophages to confer functional benefits [40]. Overall, Xin et al. developed a local exosome chitosan-based delivery system that could promote endometrium regeneration and fertility restoration [40]. They found that this system involved a mechanism-of-action related to M2 macrophage polarization with miRNA-223-3p serving as a top key component within exosomes [40].

### 5.5. Hyaluronic Acid

Hyaluronic acid (HA) is a linear polysaccharide with repeating units of D-glucuronic acid and *N*-acetyl-D-glucosamine [90]. HA is characterized by a strong water binding ability and is found in the extracellular matrix [90]. HA has been used to deliver anti-bacterial drugs [99], immunosuppressants [100], anabolic and anti-inflammatory compounds [70,101,102,103,104]. The delivery of chemotherapeutics using hyaluronic acid have been well-studied [105,106,107,108]. Examples of these delivered therapeutics include doxorubicin [109,110,111,112], cisplatin [113], and cantharidin [114]. Additionally, anti-oxidants [115], flavonols [116], anti-photoaging agents [117], and peptides [118] have been delivered using HA. Furthermore, HA has been used to deliver cells (including cell secretome) [119,120], genetic material [112], growth factors [102,121], and hormones [122].

Hyaluronic acid-based scaffolds have been used to deliver EVs as a therapy for tendon repair [45] and osteoarthritis cartilage injuries [46]. K. Song et al. isolated exosomes from tendon derived stem cells (TDSC-Exos), loaded a hyaluronic acid scaffold with the exosomes, and studied the therapeutic effects of this system for tendon repair. The authors examined exosome size distribution, morphology, and the presence of exosome-related markers (CD9, CD63, CD81, and TSG101) using Western blot [45]. They also used the protein concentration of exosomes as a representation of exosome concentration throughout the study [45]. The authors then studied the effects of TDSC-Exos on the proliferation and function of tenocytes in vitro and found that high concentrations of exosomes (100 μg/mL) could protect tenocytes from oxidative stress and serum deprivation [45]. Additionally, the treatment of tenocytes with TDSC-Exos increased type 1 collagen production and elevated tendon-specific marker (*Scx, Col1a1,* and *Dcn*) expression [45]. The authors then produced a scaffold by irradiation, containing a uniform exosome distribution that could serve as a sustained exosome-release system (50% of exosomes retained after 14 days) [45].

Song et al. then created a rat tendon defect model and used the hyaluronic scaffold (pHA) or the hyaluronic scaffold containing exosomes (pHA-TDSC-Exos) to fill the gap in the patellar tendons [45]. Not only did they see decreased wound visibility with the pHA-TDSC-Exos group, but the authors found through H&E staining that the wound healing outcomes of this group were significantly better than the pHA and control groups and could enhance tendon repair in this model [45]. Masson trichrome staining showed better collagen fiber arrangement with the pHA-TDSC-Exos group, and immunohistochemistry was used to show that this group promoted the early repair of the injured tendon with earlier type III collagen presence and reduction during the tendon healing process compared to the other groups [45]. Furthermore, pHA-TDSC-Exos facilitated the restoration of the biomechanical properties of the injured tendon [45].

With increasing evidence of the important roles miRNAs play during tissue repair, Song et al. performed RNA sequencing to find miRNAs that were expressed significantly higher in TDSC-Exos compared to tenocytes and chose to focus on miR-144-3p [45]. Song et al. showed that miR-144-3p enhanced cell proliferation and migration. They found that miR-144-3p from TDSC-Exos played an important role in tendon repair by targeting ARID1A in tenocytes [45]. ARID1A is a key component of the switch/sucrose non-fermentable ATP-dependent chromatin-remodeling complex, which plays a critical role in cell cycle modulation [45]. MiR-144-3p is only one potential mechanism by which TDSC-Exos may exert therapeutic effects [45]. Overall, Song et al. showed that TDSC-derived exosomes could promote tendon repair and that miR-144-3p transferred from these exosomes enhanced tenocyte proliferation and migration by targeting ARID1A.

### 5.6. Gelatin

Gelatin is a widely used natural biopolymer in regenerative medicine and tissue engineering [123]. Similar to the natural biopolymers discussed above, gelatin has been utilized to deliver various types of drugs including anti-fungal/anti-yeast drugs [124], anti-inflammatory agents [125,126,127], antibiotics [128,129,130], and chemotherapeutics [131,132,133,134]. Other factors such as probiotics [135], vitamins [136], peptides [137], and ions [138,139] have been delivered using gelatin. Furthermore, researchers have used gelatin to deliver cells [140,141,142], signaling molecules [143,144], and genetic material [145,146].

Gelatin-based scaffolds have been utilized for bone regenerative purposes [44]. Man et al. epigenetically enhanced osteoblast-derived EVs with the histone deacetylase inhibitor Trichostatin A (TSA) to promote EV osteoinductive potency [44]. In this study, Man et al. used gelatin methacryloyl (GelMA) functionalized with synthetic nanoclay laponite (LAP) (GelMA-LAP), which binds, stabilizes, and improves biofactor retention [44]. They characterized these enhanced EVs (TSA-EVs) using TEM imaging, nano-flow cytometry, single-particle phenotyping, measuring EV protein content, and EV release kinetics from the GelMA-LAP hydrogel [44]. The authors also found that the TSA-EVs released from the GelMA-LAP hydrogel were internalized by hBMSCs, and these EVs promoted hBMSC proliferation and migration [44]. Furthermore, the authors found that these released TSA-EVs enhanced histone acetylation and mineralization of hBMSCs [44]. Man et al. went further to evaluate the effects of TSA-EVs on hBMSC extracellular matrix mineralization within the GelMA-LAP hydrogels by assessing the ALP activity, collagen production, and calcium deposition [44]. There was enhanced ALP activity in the hBMSCs within the TSA-EV hydrogels compared to the untreated osteoblast-EVs (MO-EV) and EV-free groups [44]. hBMSC collagen production within the hydrogel was evaluated through picrosirius red staining, and TSA-EV treatment resulted in the greatest collagen content with a high dosage of TSA-EVs (50 µg/mL) (TSA-EV-50), resulting in the greatest collagen production [44]. Furthermore, TSA-EV-50 gels resulted in greater calcium deposition compared to other groups, as found through alizarin red staining [44].

Man et al. believe that the osteoinductive effect of TSA-EVs within the GelMA-LAP hydrogel is due to the 3D matrix (rather than a 2D matrix). More specifically, the 3D matrix elicits an altered cellular response to chemical and physical stimulation, so hBMSCs within the GelMA-LAP hydrogels may be more receptive to osteoinductive stimulation, which is induced by TSA-EVs compared to hBMSCs in 2D culture [44]. Additionally, the 3D microenvironment of the GelMA-LAP hydrogel may have altered the epigenetic landscape of encapsulated hBMSCs, which may have primed the cells with enhanced differentiation capacity compared to the 2D cultured cells [44]. Finally, the 3D microenvironment of the hydrogel and ECM produced by the hBMSCs likely influenced the sequestering of bioactive factors (e.g., EVs) within the secretome, which would further facilitate mineralization within the GelMA-LAP hydrogel [44]. Altogether, these findings demonstrate the improved therapeutic potential of epigenetically enhanced EVs delivered via a gelatin-based scaffold for bone regeneration.

### 5.7. Natural Biopolymer Composite Scaffolds

To help overcome issues regarding the mechanical properties of scaffolds, researchers have combined biopolymers to create composite scaffolds. Collagen plays a structural support role in wound healing and also controls cellular functions such as cell shape and differentiation, migration, and some protein synthesis [32]. However, collagen typically has poor mechanical performance, in that it has low strength, and is degraded within days at body temperature [32,147]. Abolgheit et al. used a chitosan-collagen scaffold seeded with bone marrow-derived mesenchymal stem cells (BM-MSCs) or the EVs secreted by BM-MSCs and examined skin wound healing. By cross-linking chitosan into the scaffold, Abolgheit et al. were able to obtain a scaffold with mechanical properties, such as pore size and distribution, similar to that of soft tissues [32]. Furthermore, chitosan has bactericidal properties, is biocompatible, cytocompatible, and is cost-effective [32].

Abolgheit et al. seeded BM-MSC EVs into collagen-chitosan scaffolds, however, the exact method of EV loading was not described, and EV loading efficiency was not examined. Furthermore, they did not examine EV functionality in vitro in this study. Nevertheless, they demonstrated that their scaffolds laden with MSCs or EVs showed enhanced macrophage count [32]. Furthermore, greater amounts of collagen deposition with better alignment was observed with scaffolds containing EVs compared to scaffolds with MSCs, demonstrating the advantages of using EVs rather than stem cells [32]. Abolgheit et al. found that it was the scaffold itself that was responsible for accelerated wound healing while the addition of EVs to the scaffold positively impacted the quality of wound healing. This study emphasizes the great effect that EVs may have on remodeling and wound healing, especially when incorporated into a natural biopolymer-based scaffold.

In this section, we highlighted the therapeutic advantages of incorporating EVs into natural biopolymer-based scaffolds. Some of the demonstrated advantages of EV use include improved outcomes after myocardial infarction, bone healing, and wound healing. Challenges associated with natural biopolymers such as a lack of mechanical support and reproducibility may be addressed through the production of composite scaffolds. It is clear that natural scaffolds embedded with EVs are a promising strategy for a multitude of therapeutic applications.

## 6. Synthetic Biopolymer Scaffolds for Therapeutic EV Delivery

The use of synthetic biomaterials addresses concerns that are associated with natural biomaterials such as inconsistent starting material and sterility. However, synthetic biopolymer-based scaffolds come with their own disadvantages. The mechanical properties of synthetic biopolymers may differ from natural biopolymers or tissues in terms of stiffness and elasticity, and they may also lack biocompatibility and biochemical cues (e.g., protein motifs and release of bioactive peptides) that are characteristic of natural biopolymers [148]. Despite these disadvantages, synthetic biopolymers can also be modified to reach various therapeutic goals. More specifically, the use of synthetic biopolymers allows for greater control over characteristics of the material including the chemistry, charge, and stiffness [149]. Researchers have studied a wide array of synthetic biopolymers including polyethylene glycol (PEG), polycaprolactone (PCL), poly(lactic-co-glycolic acid) (PLGA), poly(L-lactide) (PLLA), and poly(lactic acid) (PLA). Some of the benefits of utilizing synthetic biopolymers is increased batch-to-batch consistency and greater control over the physical and chemical properties of the material. Various synthetic biopolymer-based scaffolds have been utilized for EV delivery to improve healing and disease states (Table 1). Here, we discuss these therapeutic applications of EV-laden synthetic scaffolds in addition to factors that have been delivered using common synthetic biopolymers.

### 6.1. Polyethylene Glycol (PEG)

PEG is an FDA-approved, hydrophilic, and flexible polymer that has been proven to be safe for use in biomedical applications [150]. PEG has been used to deliver chemotherapeutics [150,151,152,153,154,155,156,157,158,159], anti-inflammatory drugs [160], antibiotics [161,162], and bisphosphonates [163]. Additionally, vitamins [164], phenols [165], and hormones [166] have been delivered using PEG. Furthermore, PEG has been utilized for cell [167], signaling molecule [143], and genetic material delivery [168,169,170].

Exosomes have been delivered via PEG hydrogels for cutaneous wound healing [47,171]. Additionally, macrophages adopt polarization states in response to the local microenvironment [47]. Based on Kwak et al.’s miRNA-sequencing data, exosomes derived from M1 macrophages (M1-Exos) and M2 macrophages (M2-Exos) contain proteins and miRNAs that are capable of shifting macrophage polarity [47]. Thus, Kwak et al. utilized M2-Exos to induce the reprogramming of nearby proinflammatory M1 macrophages toward an anti-inflammatory M2 phenotype [47]. They then encapsulated the M2-Exos in hydrolytically degradable PEG hydrogels (M2-Exogel) and found that the degradation time was adjustable from 6 to 27 days through controlling the crosslinking density and tightness [47].

Kwak et al. used a full-thickness excisional wound model to assess the therapeutic effects of the M2-Exogel in vivo. They treated the wounds with saline, hydrogel alone, free exosomes, or M2-Exogel. Using immunohistochemistry and cytokine expression analyses, Kwak et al. showed the successful local transition of M1 macrophages to M2 macrophages within the lesion for more than 6 days [47]. The M2-Exogel served as a long-term supply of the critical concentration of exosomes needed to initiate and sustain the reprogramming of M1 to M2 macrophages, ultimately contributing to improved wound healing [47]. Kwak et al. found that localizing M2-Exos in the hydrogel led to rapid wound closure and increased healing quality compared to other groups [47]. More specifically, the wound size significantly decreased after day 8 in the M2-Exogel group compared to other groups [47]. Furthermore, they stained closed wound tissues with Masson’s trichrome and saw that the M2-Exogels produced superior results in the stable closure of full-thickness skin wounds as well as enhanced the dermal adipogenesis and hair follicle regeneration compared to freely injected exosomes [47]. Altogether, the PEG hydrogel-based exosome delivery system serves as a method to locally regulate the polarization state of macrophages, which is critical for tissue homeostasis and proper wound repair [47].

### 6.2. Polycaprolactone (PCL)

PCL is a linear, hydrophobic, aliphatic polyester with high mechanical strength and is biocompatible as well as biodegradable [172,173]. Chemotherapeutics [154,157,159,174,175,176,177,178,179], antimicrobials [162,180,181], and anti-inflammatory drugs [160,182] have been delivered using PCL. Furthermore, PCL has been used for the delivery of hypotensive agents [183], protease inhibitors [184], anticonvulsants [172], and sulfonylureas [185]. In addition to drug delivery, PCL has been used to deliver polyphenols [186], antiretrovirals, and hormones [187]. Similar to the previously discussed biopolymers, the delivery of genetic material [169,188,189], growth factors [190], and cells [191] using PCL has been studied.

Synthetic biopolymer-based scaffolds containing EVs have been used to improve vascular performance and functionality (Table 1). Wei et al. were interested in using heparin-functionalized vascular PCL grafts to enhance anti-thrombogenicity. The authors fabricated the tubular PCL grafts using electrospinning, modified the grafts with heparin, and loaded MSC-derived small EVs (MSC-sEVs) by soaking the scaffolds in sEV solution [49]. The authors observed a uniform distribution of sEVs on the grafts using a confocal laser scanning microscope [49].

Wei et al. examined the in vivo stability of the grafts by in vivo imaging using labeled sEVs and found that the bioluminescence intensities were higher in the heparinized scaffold group [49]. The authors then studied the performance of the PCL vascular grafts modified with heparin and loaded with sEVs in a hyperlipidemia rat model [49]. sEVs play many roles in this model. Wei et al. found that heparin enhanced anti-thrombogenicity while the addition of immunomodulatory sEVs inhibited thrombosis and calcification, which therefore improved the patency of the graft [49]. The patency rate was measured using color doppler ultrasound, and H&E and von Kossa staining was used for calcification detection [49]. Additionally, bioactive molecules (e.g., VEGF and miRNA 126) from the MSC-sEVs enhanced endothelium and vascular smooth muscle regeneration, shown through H&E staining and immunofluorescence staining with the CD31 antibody, α-SMA antibody, and myosin heavy chain [49]. Furthermore, flow cytometric analysis revealed that sEVs induced polarization from pro-inflammatory and atherogenic M1 macrophages to anti-inflammatory and anti-osteogenic M2c macrophages [49]. This study further highlights how synthetic biomaterials can be enhanced using EVs to create translational scaffolds for regenerative medicine. 

### 6.3. Poly(Lactic-Co-Glycolic Acid) (PLGA)

PLGA is a copolymer that is similar to PCL in that it is a biocompatible, biodegradable, and flexible biopolymer [192,193]. The delivery of antibiotics [194,195,196,197,198] and chemotherapeutics [199,200,201,202,203,204] using PLGA has been well-studied. Dopamine agonists [205], anticonvulsants [206], statins [207], and immunosuppressants [208] have also been delivered using PLGA. Furthermore, growth factors [209], various proteins [210,211,212], hormones [213,214], genetic material [215], and vaccines [216] have been delivered using PLGA.

PLGA scaffolds containing EVs have been studied to improve bone defects [52] and chronic kidney disease [51]. Ko et al. designed a PLGA-based scaffold to deliver stem cell-derived EVs for kidney regeneration. The composite scaffold was composed of PLGA, magnesium hydroxide, and decellularized porcine kidney extracellular matrix, and polydeoxyribonucleotide (PDRN) and was fabricated using ice particle leaching [51]. The scaffold was enhanced with EVs that were derived from TNF-α/IFN-γ-primed UC-MSCs (TI-EVs) [51]. Ko et al. characterized the EVs by shape and size. They then studied the effector molecules within the EVs; more specifically, Ko et al. focused on proteins within TI-EVs and unprimed EVs (UC-EVs). The results indicated that treating UC-MSCs with TNF-α/IFN-γ enhanced the cellular uptake capabilities of secreted EVs and induced changes in protein cargo, which is indicative of kidney tissue regeneration [51].

In a partial nephrectomy mouse model, the scaffold containing PDRN and EVs induced glomerular regeneration and the restoration of kidney function [51]. More specifically, the kidney developmental factors (Pax2, Wt1, and Emx2) increased in expression with PME/PDRN/TI-EV scaffold treatment [51]. Additionally, there was an increase in the population of Pax2-expressing host cells, which indicates that the scaffold can facilitate host renal stem/progenitor cell infiltration [51]. Furthermore, there was an increased expression of pro-angiogenic growth factors (FGF2, HGF, and VEGF) in the PME/PDRN/TI-EV scaffold group [51]. Ko et al. determined the total number of functional glomeruli using H&E staining, with the PME/PDRN/TI-EV group showing the best results [51]. They then examined renal function recovery by evaluating the serum creatinine and blood urea nitrogen (BUN) levels. They found significantly better metabolic function in the PME/PDRN/TI-EV group [51]. Furthermore, the glomerular filtration rate (GFR) was restored in the PME/PDRN/TI-EV group (227.2 μL/min) to a level similar to that of native mice (232.5 μL/min) [51]. These results show structural and functional kidney tissue regeneration with the use of the PME/PDRN/TI-EV scaffold. Overall, the biochemical cues from TI-EVs and PDRN as well as the biophysical cues from the PLGA scaffold serve as potential tissue engineering platforms for kidney tissue regeneration [51].

### 6.4. Poly(L-Lactide) (PLLA)

PLLA degrades by nonenzymatic hydrolysis and its by-products are eliminated via normal cell metabolism [217]. Thus, it is biodegradable and biocompatible. The delivery of antibiotics [218,219,220,221] and chemotherapeutics [222,223,224,225,226] using PLLA has been frequently studied as well as anti-inflammatory drugs [227], anti-psychotics [228], acetylcholinesterase inhibitors [229], and ocular disease therapeutics [230,231]. In addition to drug delivery, cells [232,233], growth factors [234,235,236,237], and genetic material [238] have been delivered using PLLA. Furthermore, PLLA has been utilized to deliver hormones and fertilizer [239].

Swanson et al. engineered a biodegradable PLLA-based delivery platform to control the release of exosomes from microspheres to promote craniofacial bone healing. More specifically, they used PLGA and PEG triblock copolymer microspheres to encapsulate and control the timed release of human dental pulp stem cell (hDPSC)-derived exosomes [54]. This delivery platform was integrated with a 3D tissue engineered PLLA scaffold. They found that microspheres containing exosomes demonstrated a linear and consistent release profile over a longer period of time when attached to a PLLA scaffold compared to freely suspended microspheres [54]. They also found through NTA and TEM that the exosomes maintained their characteristic diameter and morphology throughout their encapsulation and release [54]. Furthermore, Swanson et al. confirmed the in vitro bioactivity of the exosome-containing microspheres by culturing mouse BMSCs on nanofibrous scaffolds functionalized with the microspheres. They used the colorimetric calcium assay to examine hydroxyapatite mineralization and energy dispersive X-ray spectroscopy (EDX) to determine the spatial distribution of elements in the construct [54]. It was found that microsphere-functionalized constructs and groups treated with exogenous exosomes showed an increased calcium phosphate content and a decreased proportion of organic components as minerals was deposited [54].

Furthermore, the authors demonstrated exosome functionality in vivo by implanting constructs in mice and subsequent staining (Masson’s Trichrome and von Kossa staining). This revealed that constructs with exosome-containing microspheres increased hECM deposition and promoted early mineralization compared to blank microsphere constructs and blank scaffolds, which attributes the function to the exosomes [54]. Furthermore, the constructs were used in a calvarial bone defect model. After 8 weeks, the functionalized constructs were laden with cells, collagen-rich matrix, marrow-containing bone tissue, and were integrated with the host at 8 weeks [54]. Additionally, the µCT of the skulls showed that localized delivery of the exosomes via microspheres in the scaffold resulted in the best regenerative outcome among the treatment groups [54].

Overall, the functionalized scaffold system was able to recruit endogenous cells and stimulate bone tissue neogenesis in vivo [54]. The exosomes used in this study provided pro-mineralization cues that guided local progenitor cells toward osteogenic differentiation both in vitro and in vivo [54]. By incorporating exosomes into a synthetic biopolymer-based scaffold, researchers can work toward overcoming the inherent lack of biochemical cues in synthetic biomaterials to achieve therapeutic effects such as bone healing.

### 6.5. Poly(Lactic Acid) (PLA)

PLA is biocompatible and biodegradable via hydrolysis and enzymatic activity and is also highly hydrophobic [240]. PLA has a wide range of mechanical and physical properties. Similar to the other discussed synthetic biopolymers, the delivery of antibiotics [241,242,243] and chemotherapeutics [240,244,245,246,247,248,249,250,251] using PLA has been well-studied. Additionally, therapeutics for fibrosis [252], arthritis [253], and osteoporosis [254] have been delivered using PLA. Furthermore, PLA has been used to deliver cells [255], growth factors [256], genetic material [257,258], and hormones [259]. Finally, polysaccharides [260], peptides [261], and plant-derived compounds [262,263] have been delivered via PLA.

The physical properties of PLA can be tailored, and PLA has already been used in other materials such as sutures, stents, and in oral surgery [48]. Calcium silicates (CaSi) have been shown to stimulate new bone formation in bone defects [48]. When doped with CaSi, PLA scaffolds have shown high values of bulk porosity, adequate thermal-mechanical properties, and can release Ca^2+^, OH^−^, and nucleate apatite [48]. Gandolfi et al. aimed to develop a mineral-doped PLA-based scaffold functionalized with EVs to improve osteogenic commitment of human adipose-derived MSCs. Gandolfi et al. saw that mineral-doped PLA scaffolds adsorbed red-labelled human adipose mesenchymal stem cell (hAD-MSC)-derived exosomes [48]. The exosomes were then released and internalized by the cultured hAD-MSCs, and the osteogenic commitment and properties of the hAD-MSCs were confirmed by examining the gene expression of hAD-MSCs; the examined genes included Collagen type 1, Osteocalcin, and Runx [48]. Overall, mineral and exosome-doped PLA-based porous scaffolds provided a suitable bone-forming microenvironment, triggered the osteogenic commitment of the cells, and improved the osteogenic properties of the cells [48]. As a result of the minerals and exosomes providing osteogenic cues, this scaffold system has potential in regenerative bone healing. Future work for Gandolfi et al. includes implanting this functionalized scaffold in vivo to study the potential pro-osteogenic effects on MSCs present at the wound site [48]

In this section, we examined the therapeutic advantages using synthetic biopolymer-based scaffolds endowed with EVs. Some of the demonstrated benefits of EV incorporation are enhanced wound healing, bone tissue neogenesis, kidney regeneration, and improved vascular performance and functionality.

## 7. EV Delivery via Scaffolds for Tissue Repair

Given the complex nature of the various systems and tissues that make up a living organism, it is crucial that tissue engineers design scaffolds that can restore, maintain, or improve specific tissues or whole organ function. In other words, scaffold-based delivery systems must be tailored to achieve a specific function with consideration of the natural tissue and environment that is being mimicked. As a result of the wide range of available biopolymers and the necessity for specificity in tissue engineering, there are many different engineered delivery systems for EVs. The use of biopolymers functionalized with EVs provides a novel method to address and aid tissue repair in various systems or organs. Below, we highlight the diversity of tissues that may benefit from EV delivery via biopolymer scaffolds to improve tissue repair. Furthermore, we briefly examine examples or considerations for EV delivery via scaffolds within each discussed tissue type.

### 7.1. Nerve Repair

The central nervous system (CNS) is notorious for limited regenerative capacity. Additionally, due to the high prevalence of CNS conditions (e.g., stroke), it is crucial to develop therapies to restore function to pre-injury levels [264]. It is important that the developed scaffolds mimic the CNS microenvironments in terms of viscoelasticity to facilitate the migration and differentiation of endogenous stem cells within the scaffold [264]. An example of a promising delivery vehicle is PLLA scaffolds coated with collagen IV, which have been shown to serve as excellent matrices for astrocytes [265]. This scaffold may prove useful for EV delivery in the future. Another consideration is incorporating EVs into hydrogel scaffolds to maximize the therapeutic potential of EVs in intracerebral administration [264]. Furthermore, exosomes have been delivered from a fibrin gel to accelerate recovery from spinal cord injury [266]. VGF (nerve growth factor inducible) was abundant in the exosomes used in this study and played a key role in increased oligodendrogenesis in vitro and in vivo, thus aiding in functional recovery. It is expected to see a greater development of EV delivery via scaffolds for neural repair in the future.

### 7.2. Bone Repair

Bone tissue is dynamic and has a regenerative capacity, as seen through bone remodeling [267]. However, the bone regenerative process may become impaired due to infections, genetic disorder, trauma, etc. and require bone grafting [267]. Conventional autologous bone grafts are relatively easy to obtain and include osteogenic, osteoinductive, and osteoconductive properties [268]. Additionally, autologous bone grafts do not raise an immune response nor do they transmit infectious diseases as the graft is the host’s own tissue [268]. However, autologous bone grafts require an additional operative time, can be difficult to mold to the receiving site, and are associated with donor site morbidity [269]. Furthermore, the volume of bone that can be harvested from a site is limited, and transplanted bone may resorb [270]. With increasing evidence of EV-laden scaffolds aiding in bone healing, it may be possible to design a biomaterial that could replace autologous bone grafts [48]. This would avoid donor site morbidity, provide needed bone volume, and likely reduce costs.

Biopolymers for bone regenerative purposes that have been well-studied include ceramic, polymer, and composite materials [270]. Typically, scaffolds for bone regeneration utilize organic and inorganic biomaterials due to bone tissue naturally containing inorganic and organic phases [270]. EVs can be tailored or engineered for purposes such as bone regeneration. For example, osteoblast-derived EVs have been shown to promote bone marrow-derived mesenchymal stem cell differentiation into osteoblasts [270]. Some other designs include specifically engineering EVs to enhance osteogenic differentiation [271] and creating irregular scaffolds to better reflect the microstructure of the cortical/cancellous bone unit [272]. Ultimately, there are numerous approaches and methods to improve bone regeneration, and integrating EVs with scaffolds is one of the most promising avenues with excellent translational potential.

### 7.3. Cardiovascular Tissue Repair

When it comes to the cardiovascular system, heart failure is one of the worldwide leading causes of death [273]. Heart transplants are the standard curative therapy for end-stage heart failure, however, donor shortages and the requirement of lifelong immunosuppression after organ transplantation drives the field of cardiovascular tissue engineering [273]. One of the main goals of cardiovascular tissue engineering is developing regenerative grafts/scaffolds to restore lost cardiac tissue. An example of this is using tubular scaffolds to bypass or replace defective arterial segments [33]. Another example incorporates MSC-derived EVs into a cardiac scaffold for local and systemic immunomodulation following myocardial infarction [274]. Furthermore, small EVs may be incorporated into alginate scaffolds for delivery and retention in the heart, thus improving the therapeutic effects of the small EVs [26]. EV incorporation into such scaffolds works toward restoring lost cardiac tissue.

### 7.4. Wound Healing

The regenerative effects of EVs in terms of wound healing have been explored. The prevalence of combat, burn, and diabetic wounds demonstrate the need for therapies that facilitate wound healing. Exosomes can promote the proliferation and migration of fibroblasts as well as regulate Type I and III collagen and fibronectin expression [275]. Furthermore, chitosan-based hydrogels enriched with exosomes have shown positive effects on wound healing by promoting angiogenesis and tissue granulation formation [35]. Additionally, Zhao et al. found that gelatin methacryloyl (GelMA) hydrogels embedded with exosomes showed accelerated re-epithelialization, collagen maturity promotion, and improved angiogenesis in vivo [276]. Wound healing approaches that deliver EVs via scaffolds have been heavily studied and have a lot of translational potential [32,37].

## 8. EV Delivery via Scaffolds for Immunomodulation

The immune cells and mediators play important roles in mediating tissue homeostasis and reparative processes through a variety of orchestrated events and by being involved in inflammation, angiogenesis, and stem/progenitor cell activities such as proliferation and differentiation [277,278,279]. Similar to MSCs that have immunomodulatory functions, the immunomodulatory function associated with MSC-exosomes/EVs involves promoting the M2-like macrophage phenotype, Treg population, and TH2 immune responses [280,281]. Both immunomodulatory and proangiogenic factors were detected in MSC exosomes through proteomic profiling [282]. It was reported that most of the cells absorbing MSC-derived exosomes from the scaffolds were immune cells [282]. This may imply the importance of immune responses in tissue reactions to scaffolds with and without EVs and the necessity to understand and use this mechanism to guide the biological communication network between different cell types. In addition, the cellular communication network between the immunomodulation and regeneration processes in exosome-functionalized scaffolds still needs to be elucidated in detail.

Inflammation is indispensable and irreplaceable in the process of tissue repair and both positively and negatively regulates the tissue repair process. The tissue repair cascade starts from a pro-inflammatory reaction and must be regulated in a timely manner to ellicit appropriate tissue repair/regeneration. Along this line, MSC exosome–functionalized scaffolds were shown to induce innate and adaptive immunomodulatory responses toward tissue repair by proactively recruiting immune cells and modulating orchestrated M2/TH2/Treg responses locally and on the systemic level [282]. In addition, three-dimensional-printed scaffolds consisting of bioceramic-induced macrophage exosomes were demonstrated to regulate immunomodulation, osteogenesis, and angiogenesis [283].

## 9. Challenges in EV Loading, Integrity, Delivery, and Scaling Up

Despite the positive therapeutic effects of EVs, challenges regarding the uniform EV loading of scaffolds remain. More specifically, EV or secretome solutions may not reach the innermost regions of scaffolds depending on the EV loading method [284]. For example, Bari et al. showed that the lyosecretome solution did not reach the innermost regions of their 3D printed PCL scaffold when an adsorption loading method was used [284]. Additionally, this simple adsorption method was accompanied with high batch-to-batch loading variability [284]. However, Bari et al. utilized a second loading method involving co-printing PCL and lyosecretome-containing alginate, which demonstrated a slower release profile as well as the homogeneous loading of proteins/lipids [284]. It remains to be elucidated whether this second method reduces the batch-to-batch loading variability. Each of these methods are useful for specific goals (e.g., quick or prolonged EV release), however, adsorption loading methods may result in EV loading variability.

In another study, Xing et al. modified a silk fibroin/PCL scaffold with polydopamine (pDA). They found through laser scanning confocal microscopy that pDA modification of the scaffold led to significantly more efficient EV loading compared to conventional superficial EV adsorption onto the silk fibroin/PCL scaffold [285]. Xing et al. also found that the EV adsorption technique led to a burst release effect with EV depletion within nine days [285]. Meanwhile, in the pDA-modified scaffold, EVs demonstrated a slow-release profile with about 10% of EVs retained within the scaffold after nine days [285]. These studies highlighted overcoming challenges regarding EV loading efficiency and sustained EV release from the scaffolds. Researchers may want to consider scaffold loading alternatives to simple EV adsorption such as unique 3D printing scaffold design or biopolymer modification to overcome the discussed challenges.

An important concern regarding EV delivery is the maintenance of EV lipid membrane integrity. The lipid membrane plays an important role in protecting the nucleic acid cargo of EVs, which is responsible for the physiological effects of EVs [54]. Thus, it is crucial that the lipid membrane of EVs remains intact throughout scaffold loading and delivery. EV membrane integrity may be evidenced through electron microscopy (although this method is not high-throughput) or through assays that examine proteins that are tethered to the EV membrane (e.g., CD81) [286]. Furthermore, EV integrity may be studied by examining the EV characteristics prior to scaffold loading and after release from the scaffolds. For instance, Huang et al. found that engineered EVs maintained their integrity before scaffold loading and after delivery [20]. This was demonstrated through nanoparticle tracking analysis (NTA) and polydispersity index (PDI) in which EVs released from RGD peptide-containing hydrogels maintained a similar size distribution and PDI compared to the pre-scaffold encapsulation control group [20]. Following this confirmation, they found that the EVs maintained their desired functionality in vitro and in vivo after hydrogel encapsulation [20].

Nevertheless, there is a need for more widespread confirmation of maintained EV membrane integrity and bioactivity. For instance, Su et al. loaded and tethered exosomes using polyethylenimine (PEI) molecules to a PCL-based scaffold [282]. They found that the exosomes released from this scaffold did not lose their function regarding modulating macrophages to M2-like phenotypes in vitro compared to freely suspended exosome vesicles [282]. Even though Su et al. characterized the diameter and zeta potential of exosomes before scaffold loading, they did not report the examination of these same parameters following exosome release from the scaffold. Many studies such as [287,288] characterized EVs prior to scaffold loading and went on to examine the in vitro and in vivo bioactivity of the loaded scaffolds. These studies failed to confirm the physical integrity of the EVs when released from the scaffolds. Overall, many current approaches have demonstrated positive bioactive effects of EVs delivered from the scaffolds, however, few studies have confirmed EV membrane integrity after delivery. It is important that researchers take this extra confirmation step so that they can attribute the observed functionality to EVs, thus strengthening and supporting their findings.

Furthermore, there are issues concerning the optimal scaffold pore size and pore size distribution of the scaffolds. Pore parameters play a role in controlling the release of EVs, and the pore size contributes significantly to the effects the microenvironment has on cellular functions (e.g., cell adhesion and polarization) [289,290]. Additionally, the average pore size affects the rheological phenotype and diffusivity of the scaffolds [289]. Furthermore, the distribution of pore sizes in a scaffold affects the analysis of the mechanical properties of the scaffold, diffusive processes, and cell migration through the scaffold [289]. There is no agreement on the optimal porosity value or pore size, however, as long as mechanical properties of a scaffold are satisfied, porosity values over 90% are recommended, along with a pore size range from 10 to at least 200 μm [48].

Other challenges concerning scaffolds include the consistency of produced scaffolds and scaling up production. When it comes to scaling up efforts, engineered scaffolds may work well at a small scale, however, it may be difficult to scale up these materials for larger trials, unless the production of each type of unique scaffold is standardized. Furthermore, EV isolation may also need to be standardized to allow for consistency and reproducibility, especially for scaling up measures.

## 10. Conclusions

In this review, we discussed the important roles EVs play and their therapeutic potential. We also explored the different aspects of natural and synthetic biopolymers and how EVs may be incorporated into these scaffolds. Delivering EVs bypasses the concerns regarding the use of stem cells in tissue engineering. As evidenced by the discussed studies, scaffolds functionalized with EVs have a wide range of applicability including tissue remodeling, wound healing, and bone healing. Even though natural and synthetic biopolymer-based scaffolds each have their own advantages and disadvantages, their therapeutic potential can be enhanced by incorporating EVs. Throughout this review, we highlighted the potential mechanisms by which EVs impart therapeutic effects and their role in immunomodulation. Overall, there are many types of biopolymers that may be used to deliver EVs, and research concerning how EVs exert therapeutic effects is ongoing.

## Figures and Tables

**Table 1:** The therapeutic applications of natural and synthetic biopolymer-based scaffolds that utilize extracellular vesicles. The type of EV and biopolymer used in each study are indicated as well as the EV incorporation method into the scaffold.

Study	EV Source	Scaffold Biomaterial	Modifications and/or EV Incorporation Method	Disease or Tissue	Animal Models	Therapeutic Outcomes
[33]	Human adipose-derived mesenchymal stem cell (hADMSC) EVs	Silk-based tubular scaffold	Vacuum-seeded EVs	Cardiovascular disease	Rat abdominal aortic interposition graft model	Improved patency and matrix deposition, including increased elastin and collagen production
[26]	Bone marrow-derived mesenchymal stem cell (BMSC) sEVs	Sodium Alginate	sEVs mixed with sodium alginate solution	Myocardial infarction (MI)	MI induction in male rats	Decreased cardiac cell apoptosis Promotion of macrophage polarization Increased scar thickness and angiogenesis Improved cardiac function and infarct size
[37]	Human umbilical cord mesenchymal stem cell (HUCMSCs) Exosomes	Polyvinyl alcohol (PVA)/Alginate	Exosomes mixed with PVA/alginate solution	Diabetic wound healing	Full-thickness wounds on diabetic rat model	Proliferation, migration, and angiogenesis of HUVECs Sped up diabetic wound healing by promoting angiogenesis
[38]	ADSC-derived Exosomes	Sodium Alginate	Exosomes mixed with alginate solution	Peripheral nerve regeneration	Rat sciatic nerve defect	Exosomes containing neurotrophin-3 mRNA (important neurotrophic factor for peripheral nerve regeneration) in scaffold promoted nerve regeneration
[39]	Dental pulp stem cell (DPSC)-derived EVs	Collagen	EVs were injected into the scaffold	Bone regeneration	Rat calvarial bone defect	Bone formation in center of defects Broader angiogenesis
[40]	HUCMSCs	Collagen	Exosomes added dropwise onto scaffold	Endometrium regeneration and fertility restoration	Rat endometrium-damage model	Induction of endometrium regeneration, collagen remodeling Increased expression of estrogen receptor α/progesterone receptor Restored fertility Facilitated CD163+ M2 macrophage polarization, reduced inflammation, increased anti-inflammatory responses
[32]	BMSC EVs	Chitosan-Collagen Composite Scaffold	Scaffolds seeded with EVs	Skin wound healing	Full-thickness skin wound on adult male rats	Accelerated skin healing Enhanced macrophage count Greater collagen deposition, better collagen alignment and thus, increased mechanical strength
[41]	Bone mesenchymal stem cell-derived sEVs	Chitosan	sEVs added to hydrogels	Bone defect repair	Calvarial defect rat model	sEV-loaded hydrogel promoted bone healing by enhancing angiogenesis possibly via upregulation of miR-21 in sEVs
[42]	Induced pluripotent stem cell-derived mesenchymal stem cells (iPSC-MSCs) exosomes	Chitosan	Exosomes stirred into chitosan solution	Corneal diseases	Rat cornea anterior lamellar damage model	Promote repair of damaged corneal epithelium and stromal layer
[43]	Chondrocyte exosomes	Chitosan-Gelatin-Chondroitin Sulfate and Nano-Hydroxyapatite-Gelatin	Exosome suspension added dropwise over scaffold	Articular cartilage injuries	N/A	Enhance proliferation and migration of chondrocytes
[44]	Osteoblast-derived EVs	Gelatin Methacryloyl (GelMA)	GelMA functionalized with nanoclay laponite	Bone regeneration	N/A	Enhanced proliferation, migration, histone acetylation, mineralization of human bone marrow stromal cells
[45]	Tendon derived stem cells (TDSCs) exosomes	Hyaluronic acid	Loaded scaffold–not sufficiently described	Tendon repair	Rat model of tendon defects	Promoted proliferation, migration, collagen type I production, and tendon-specific markers expression in tenocytes Protected tenocytes from oxidative stress and serum deprivation Promoted early healing of injured tendons and better fiber arrangement at injury site
[46]	Human articular chondrocyte-derived EVs	Hyaluronic acid/Chitosan	Loaded scaffold–not sufficiently described	Osteoarthritis cartilage injuries	Rabbit osteochondral defect model	Greater cartilage regeneration Provide niche for chondrogenic differentiation of MSCs Hyalin-like cartilage in defect zone
[47]	M2 polarized macrophage-derived Exosomes (M2-Exos)	PEG	Dissolved freeze-dried PEG in exosome solution	Cutaneous wound healing	Mouse excisional wound splinting model	Localization and sustained release of M2-Exos Effective and prolonged conversion of M1 to M2 macrophages Enhanced efficiency and quality of wound care
[48]	hADMSC-derived exosomes	PLA	Mineral doped PLA scaffolds adsorbed exosomes	Bone defects including oral bone defects	N/A	Increased osteogenic commitment of MSCs
[49]	Mesenchymal stem cell (MSC)-derived sEVs	PCL	sEVs loaded onto heparin-modified scaffold	Cardiovascular disease	Hyperlipidemia rat model	Inhibited thrombosis and calcification and thus improved patency of graft Enhanced endothelium and vascular smooth muscle regeneration Induced polarization of M1 macrophages to M2c macrophages
[50]	MSC-exosomes	PCL	PCL modified with S-nitrosoglutathione (GSNO); exosomes incubated with scaffold	Bone defects	N/A	Decreased expression of pro-inflammatory genes in macrophages treated with exosome-loaded scaffold Accelerated osteogenic differentiation of mesenchymal stem cells
[51]	MSC-derived EVs	PLGA	EVs infused into composite scaffold; polydeoxyribonucleotide (PDRN) added	Chronic kidney disease	Partial nephrectomy mouse model	Synergistic interaction of EVs and other added compounds in scaffold alleviates fibrosis and inflammatory response Cellular proliferation Angiogenesis Effective glomerular regeneration Restoration of kidney function Develop new blood vessels and induces pro-reparative macrophages
[52]	Human adipose-derived stem cell exosomes	PLGA	Scaffolds submerged in exosome solution	Bone defects	Mouse calvarial defect	Enhance bone regeneration partially through osteoinductive effects and promoting mesenchymal stem cell migration and homing in newly formed bone tissue
[53]	MSC-sEVs	PEG/Hyaluronic Acid	sEVs mixed with scaffold solution	Osteoarthritis (OA)	Traumatic OA rat model	Improved bioavailability and therapeutic efficacy of MSC-sEVs for OA improvement
[54]	Human DPSC-derived exosomes	PLLA	Exosome encapsulation in triblock PLGA-PEG-PLGA microspheres and subsequently incorporated into PLLA scaffold	Bone defects	Critical size mouse calvarial bone defect	Stimulated bone tissue neogenesis Facilitated bone marrow stromal cell osteogenic differentiation Guided local progenitor cells towards osteogenic differentiation and bone healing Accelerated bone healing
